# *QuickStats*: Percentage* of Visits to Office-Based Physicians^†^ by Adults Aged ≥18 Years for Diabetes Mellitus,^§^ by Sex and Age — National Ambulatory Medical Care Survey, 2015

**DOI:** 10.15585/mmwr.mm6646a7

**Published:** 2017-11-24

**Figure:**
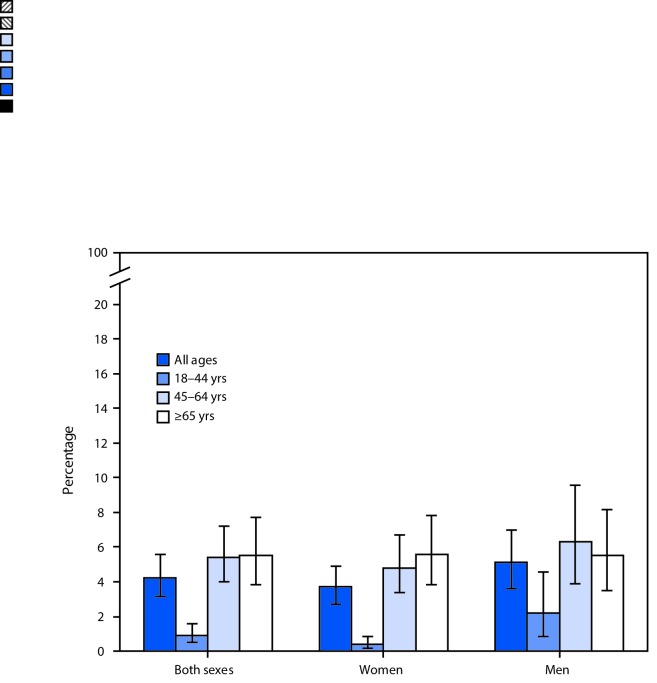
In 2015, diabetes was a reason for 4.2% of visits by adults to office-based physicians. Men aged 18–44 years had a higher percentage of visits for diabetes compared with women aged 18–44 years (2.2% versus 0.4%, respectively). Both women and men aged 18–44 years had a lower percentage of visits for diabetes compared with adults aged 45–64 and ≥65 years.

**Source:** National Ambulatory Medical Care Survey, 2015 data. https://www.cdc.gov/nchs/ahcd/ahcd_questionnaires.htm.

